# Combining Supervised and Unsupervised Learning Algorithms for Human Activity Recognition

**DOI:** 10.3390/s21186309

**Published:** 2021-09-21

**Authors:** Elena-Alexandra Budisteanu, Irina Georgiana Mocanu

**Affiliations:** Computer Science Department, University Politehnica of Bucharest, RO-060042 Bucharest, Romania; elena.budisteanu@stud.etti.upb.ro

**Keywords:** human activity recognition, skeleton, spatial-temporal graph convolutional network, clustering, k-means, Gaussian mixture model

## Abstract

Human activity recognition is an extensively researched topic in the last decade. Recent methods employ supervised and unsupervised deep learning techniques in which spatial and temporal dependency is modeled. This paper proposes a novel approach for human activity recognition using skeleton data. The method combines supervised and unsupervised learning algorithms in order to provide qualitative results and performance in real time. The proposed method involves a two-stage framework: the first stage applies an unsupervised clustering technique to group up activities based on their similarity, while the second stage classifies data assigned to each group using graph convolutional networks. Different clustering techniques and data augmentation strategies are explored for improving the training process. The results were compared against the state of the art methods and the proposed model achieved 90.22% Top-1 accuracy performance for NTU-RGB+D dataset (the performance was increased by approximately 9% compared with the baseline graph convolutional method). Moreover, inference time and total number of parameters stay within the same magnitude order. Extending the initial set of activities with additional classes is fast and robust, since there is no required retraining of the entire architecture but only to retrain the cluster to which the activity is assigned.

## 1. Introduction

The world’s population is growing rapidly. As a result, the costs of social care and hospitalization constantly increase. In order to reduce these costs, there is a need to maintain elderly people living in their homes for a long time. In order to make this possible, intensive research is conducted in the interest of creating assistive systems that are able to perform continuous monitoring of health and activity performed by elderly at home and to detect early stages of abnormal situations.

Based on a study of the World Health Organisation, assistive systems enable people to live healthily and independently, reducing the need for formal health and support services [1]. Different systems were already proposed using robotic platforms: (i) PHAROS [2,3], a socially assistive robot that monitors and evaluates the daily physical exercise performed at the user’s home; (ii) HOBBIT [4], a robot that is able to autonomously navigate around the user’s apartment, collecting objects from the ground, bringing a specific object that is requested, providing games for entertainment, and reminding the user to take their medication; (iii) RAMCIP [5], a robot that integrates several functionalities that promote physical and cognitive activity and fall detection; and (iv) a robot that integrates human activity recognition and provides assistance when required [6].

Human activity recognition (HAR) represents a challenging task for assistive robots. A HAR model for mobile robots was proposed in [7,8]. Accurate activity recognition is challenging because human activity is very complex and diverse as it depends on a series of factors not only limited to the subject’s pose or the environment in which the activity takes place but also to other physical and psychological aspects [9].

For HAR, there is a dire need for large datasets with already annotated and highly diverse samples so that neural networks can generalize well and provide high performing inference time. However, the amount of available datasets is limited, and the majority of the presented annotated data address activities related to sports or daily activities.

The motivation for this work comes from the fact that HAR comes in many forms, such as directly from RGB (Red Green Blue) videos, from RGB-D (Red Green Blue—Depth) videos, or sensor-based methods. Although all approaches are feasible and each one of them has its advantages and disadvantages, for human activity recognition, skeleton joint representation over space and time is the most remarkable descriptor of actions as it can be seen as a projection of the entire activity. Moreover, this method of representation denotes the advantage of being invariant to scene illumination, background, or cast shadow.

However, due to high computation cost, the skeleton representation proved to be a feasible solution in terms of performance and efficiency trade-off. Starting from these connections, Graph Convolutional Networks (GCNs) [10] are the most natural and most used solution for processing skeletal data. GCNs achieved remarkable results for skeleton-based activity recognition by modeling the data in such a manner that enables information flow between neighboring nodes of a graph, making it suitable for non-regular domains.

The topology of human skeleton representation is a classic graph structure, where the joints can be described as the graph nodes and the bones can be defined as the graph edges. Starting from these connections, GCN is the most natural and used solution for processing skeletal data.

Most research focuses on determining the label of each independent activity, but the connection between highly similar activities is not taken into consideration.

The present method focuses on 3D skeleton time sequences. It proposes a method that uses activity clustering as a first stage. One advantage of the clusterization consists in adding a new action (that was not previously observed) that will not be conducted to retrain the model. Then, the method is combined with a supervised method such that the entire framework is much more robust to changes of activities in the dataset.

Moreover, the proposed method tackles the problem of adding new unseen activities into a dataset, without having to retrain a heavy model. In this direction a two-stage approach was proposed: first, a discriminant model that can group activities based on their degree of similarity was used, and then a trained model is used for each of these clusters. Having a separate model for each cluster allows it to have smaller models; thus, the training will benefit from better convergence in terms of speed and accuracy. Current method defines “the hyper-label” of an action as the cluster to which the action belongs. As part of the experiments, the initial architecture was updated and a single classifier was kept instead of multiple ones by adding the hyper-label as an additional feature for the proposed model.

The paper is organized as follows: Section 2 describes the main related work models. Section 3 presents the proposed architecture along with all the experiments that were made in order to improve the performance of the proposed architecture using the evaluation of the three datasets. Section 4 contains the analysis of the performance of the proposed method and comparison results with existing methods. Conclusions and future work are provided in Section 5.

## 2. Related Work

One of the most used representations of data is provided by the human skeleton. In this direction, non-graph methods containing features obtained using neural networks are proposed. Due to the intrinsic graph structure of the skeleton, many researchers have proposed graph-based methods of representation [10,11,12]. These graph-based approaches are used in conjunction with Convolutional Neural Networks (CNN) that result in a baseline GCN.

In [13], a novel neural network architecture is proposed, and it is designed on a generic representation of skeleton sequences. Unlike other state-of-the-art approaches that rely either on the spatial configuration of the skeleton joints or on the temporal dynamics of the joints, the authors came up with a method to combine both aspects in order to fully benefit from the strength of deep neural networks. They achieved this by extending the graph representation of skeleton joints to a spatial-temporal graph model, called Spatial-Temporal Graph Convolutional Network (ST-GCN). With this model, information is integrated over both the spatial and the temporal dimension, and multiple layers of spatial graph convolutions are designed.

One advantage of the ST-GCN is that it describes convolutional architecture. Previous work [14] provides a Recurrent Neural Network approach based on a LSTM architecture which is very sensitive to noise. The authors extend their main model with Trust Gates in order to rectify the noise in the provided data and the possible occlusion of the subject. The Recurrent Neural Network manages to capture the time dependency of the activity but, in the end, achieves less performance than the ST-GCN model.

The ST-GCN model cemented as a good starting point for further improvements in the state of the art of Skeleton Based Human Activity Recognition. In paper [15], the ST-GCN model is extended to Actional-Structural Graph Convolutional Networks (AS-GCN). This extension describes the addition of an encoder-decoder submodule. In this case, instead of only capturing the local dependency of the individual joints and their local neighbors across time, the authors infer a macro dependency between distant joints, such as ‘left-palm’ and ‘left-foot’ over the course of an action. This dependency is inferred during training via structures called “A-Links” using an encoder-decoder architecture. The encoder directly models the inference of the “A-Links”, while the decoder structure predicts future poses of the skeleton.

Although the previous methods are modeled as supervised tasks, it is in our best interest eliminate supervised approaches, as data annotation can be an exhausting process.

In this direction, there are a few unsupervised systems for skeleton-based action recognition at this moment, since obtaining relevant feature representation of the skeleton can be challenging. The method Predict and Cluster  [16] proposes a method of clustering similar activities into the same cluster and different activities into separate clusters in a fully unsupervised manner. They successfully implemented a method based on an encoder-decoder recurrent neural network, which can produce a feature space in its hidden states that can effectively learn distinct actions.

Unsupervised systems for skeleton-based activity recognition are limited [17,18], since obtaining relevant feature representations from spatial representation of the skeleton is challenging. The main objective of the approach presented in this paper is to obtain an intrinsic representation of the activities in such a manner that similar activities are clustered together, while different activities are placed apart from each other, when inspecting their hidden representation. The authors proposed two decoder strategies called Fixed Weights and Fixed States in order to penalize the decoder. Predict and Cluster unsupervised system achieves high accuracy performance and outperforms the supervised methods. The hidden representation comes from the hidden states of the last layer of the encoder module, which is then fed to the Decoder submodule. The important thing is to notice how, during the training of the encoder-decoder module, the labels of the activities are not used at all, as the encoder-decoder module does not need any information about the type of activity that is performed. Keeping this in mind, in order to validate that the encoder-decoder module works well, the authors proposed encoding each activity and extracting the features from the latent space, and then they proposed useing a KNN algorithm to assign labels based on the distances between those features in latent space and the ground-truth labels, resulting in over 90% accuracy on the NTU-RGB+D dataset. On the large scale NTU-RGB+D [19] dataset, Predict and Cluster performs extremely well on the cross-view test (see Table 1). These results prove that the encoder-decoder module is a good method for representing the activities in a latent space.

Results of the existing methods are presented in Table 1.

Some of the activities are misclassified due to their similar behavior of the subject movements. The problem, in this case, is that the skeleton representation is polluted with noise due to the inaccurate detection of the joints. For example, boundary joints, such as the joints representing the feet of the subjects, encounter random movements, even if the subject is standing still. These random movements tend to signal that the activity involves movement of the lower part of the body, making the model misclassify it with another activity that actually involves movement. GCN rarely explores the effective hidden feature representation of an action. Indeed, the key features of actions can result in efficient clustering such that the categorization will be more intuitive, but the ST-GCN model alone is not able to correctly capture them.

Combining supervised and unsupervised methods has been proposed in several works. Paper [20] proposed the enhancement of an unsupervised approach in image classification. The authors state that by jointly training supervised and unsupervised cost functions using backpropagation in a neural network, one can reduce the error rate of a task when compared to individual approaches. In essence, the supervised technique is depicted as a multi-layer perceptron (MLP) or convolutional neural network (CNN), while the unsupervised one is established on a decoder submodule. A more general approach is presented in [21]. It aggregates between supervised and unsupervised techniques via unconstrained probabilistic embeddings over their ensemble. The proposal consists of conditioning the outputs of the supervised models on the constraints implied by the unsupervised ones.

The novelty of the paper consists in a new model for HAR with the following features:

1. Combines unsupervised with supervised methods for performing human activity recognition using human skeleton data (without RGB information); in fact, GCNs are combined with unsupervised techniques;
2. Has the ability of generalization for new unseen activities;
3. Has results comparable with state of the art methods (with short training time) using different augmentation techniques and hyperparameters search;
4. Uses general activities (no certain group of activities were considerred) for evaluations (activities from public datasets).

## 3. Proposed Method

**Figure 1:**
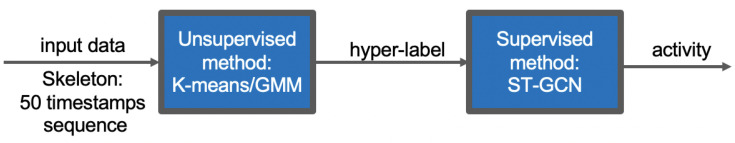
General architecture for the proposed method.

The proposed method will quantify the resemblance between different activities through their co-occurrences in the predictions of a video classification model (using the human skeleton extracted from the video). It resembles a combination of unsupervised and supervised learning algorithms, as provided in Figure 1.

The first part, the Discriminant, employs an unsupervised method of clustering data where the labels of the dataset are not used. The advantage of using unsupervised techniques is that it allows the addition of completely new, unseen samples without the need of retraining from scratch. The choice for the unsupervised algorithm is not fixed, as one can use any unsupervised clustering approach.

The second part is represented by supervised methods that require annotated datasets for training. In our case, the paradigm of ST-GCN is applied using the same structure, but instead of relying on a single model that learns the entire dataset, multiple ones can be used with extra information from the first step.

**Figure 2:**
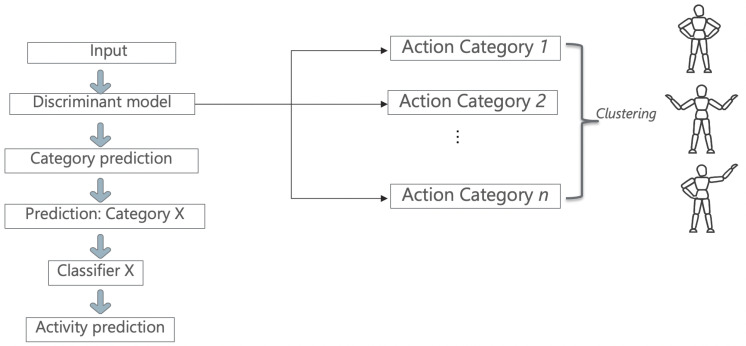
General structure of the proposed method.

Starting from this idea, a model that can discriminate between activities by grouping them together based on their similarity measures was created. The first part performs clustering that can be seen as a ‘discriminative model’. After the datum is fed into this model and a “hyper-label” is assigned to each activity, the input will be analyzed by a small ST-GCN model, which can classify only activities belonging to its subset. Rather than having a single model to generalize all the activities from the dataset, the dataset is split into multiple categories in order to reflect the similarities between them. Figure 2 depicts the architecture of the proposed method.

First, the input datum is fed to the Discriminant model, which learns how to classify each category outputted from the classification step. This prediction outputs a number between one and n (n is the number of clusters), which correspondingly picks another model to follow through with the data. This new model learns to only classify activities belonging to its category and predicts the final activity.

For the Discriminant model, the Predict and Cluster [16] method was used as an initial starting point. An encoder-decoder model is proposed in this method. The encoder-decoder architecture is based on a sequence-to-sequence Recurrent Neural Network, which proved to be very effective in modelling time dependencies of features having multiple dimensions.

The purpose of the encoder submodule is to project the input data into subsequent representations with the main property of self-organization in the latent space in order to show that the data can be clustered in an unsupervised fashion. The authors propose a bidirectional flow with the purpose of capturing better long-term dependencies of the input data. To be precise, the encoder is a multi-layered bidirectional Gated Recurrent Unit (GRU) for which its input is a sequence of joint positions corresponding to an activity. The output of the encoder submodule, specifically the last hidden state, is fed into the decoder part of the architecture.

The decoder submodule is also based on Recurrent Neural Networks, and it is implemented with a unidirectional Gated Recurrent Unit. The main purpose of the decoder is to regenerate the original sequence, starting from the hidden representation extracted by the encoder. The output of the decoder submodule is a sequence of joint positions similar to the input of the encoder.

By having both the input sequence and the output sequence, the model can be trained in an end-to-end fashion, using a regeneration loss function as an L1 loss. Note that the encoder-decoder model does not define the Activity Recognition task, as the output is another sequence of joint positions. To this end, the authors propose a validation measure in order to prove that their approach is a correct representation of the intrinsic features of the latent space where the activities are projected.

The validation measure starts with the embeddings extracted from the application of the encoder on the initial data. The embeddings represent the features of the input data as observed in the latent space. On these embeddings, the authors propose a k-Nearest-Neighbours (kNN) algorithm, with k = 1. The k-Nearest-Neighbours is fitted using the embeddings of the training data by passing the training sequences through the encoder part. After fitting the kNN, we can use the validation data by passing through the kNN and predicting the labels according to the latent space. These predicted labels are compared to the real labels, and an accuracy measure is computed based on them.

The latent space’s main characteristic is that it is able to represent the activities sequence and cluster them together based on their similarity. The kNN algorithm that was used for classification is able to group the data by choosing the closest neighbour and picking its label. However, for our unsupervised approach, we do not need the labels of the activities; intead, a more general label that should represent a broader set of activities is required. This new label becomes the initial ‘hyper-label’ described in the previous sections.

Thus, the Discriminant module is extracted from the Predict and Cluster method, which is trained independently, as a prerequisite step. After the training, the clustering algorithm is also fit on training data embeddings by using a number of N clusters.

The next step is to instantiate n classifiers, specifically ST-GCN models: one for each of the clusters used for the clustering algorithm. The training of the n clusters begins with the passing of the input data into the discriminant, which outputs the ‘hyper-label’ assigned to the activity. This ‘hyper-label’ activates the flow of data only for the corresponding classification and passes the original sequence through it, as in the normal ST-GCN.

ST-GCN is a GCN that is actually a generalization of CNNs. The biggest difference between GCN and CNN is that CNN is performing convolution on a Euclidean space (e.g., images), but GCN is performing convolution on non-Euclidean space such as graphs. Convolutions applied on spatial domains are implied to represent data as graph nodes and their connections. Then, the convolutions are applied directly on the graph nodes and their neighbors.

The output of the ST-GCN is a predicted label, which is compared to the original label using Cross Entropy Loss, which is propagated back into the corresponding ST-GCN in order to update its weights. We do not have to propagate the loss through the Discriminant, since the Discriminant model is already trained, and it is set in such a fashion as to not generate gradients in the flow of the data. The algorithm for training the model is provided in Algorithm 1.

The ST-GCN model respects the description from [13]. It starts with a Batch Normalization layer to normalize the position of the input skeleton sequence. Then the model describes nine consecutive layers of ST-GCN blocks. These nine layers are arranged sequentially, with the first three layers having an output size equal to 64, the middle three having an output size equal to 128, and the last three having an output size equal to 256. Between each layer, a dropout is applied to reduce overfit. The third, sixth and ninth layer denote a bigger stride of two in order to act as a pooling layer. At the end, a global pooling layer is used to obtain a vector containing 256 features, which is later fed to a SoftMax function for classification. Each layer of ST-GCN denotes a residual connection, except for the first layer. The temporal kernel size is set to nine over the whole network.
**Algorithm 1** Algorithm for training the proposed architecture.**N** = number of clusters**E** = encoder model**K-means** = K-means model$STGCN_{1}$, $STGCN_{2}$, …, $STGCN_{N}$ each ST-GCN model corresponds to a single cluster**for**$X_{seq}$, $Y_{label}$ in dataset **do**    $embeddings\leftarrow E(X_{seq})$    $Y_{pseodo}\leftarrow K-means.predict\left(embeddings\right)$    $Y_{pred}=STGCN_{Y_{pseudo}}\left(X_{seq}\right)$    $Error\leftarrow CrossEntropy(Y_{pred},Y_{label})$    BackPropagate Error through $STGCN_{Y_{pseudo}}$**end for**

**Figure 3:**
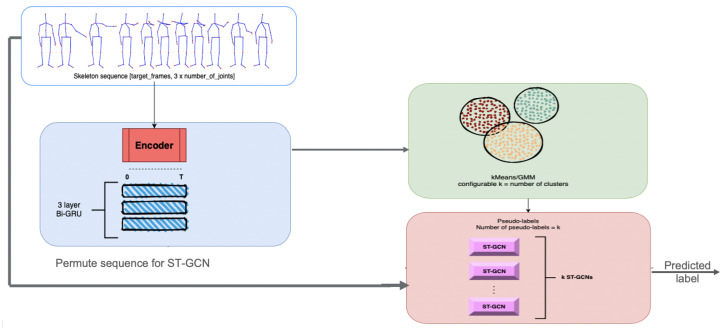
The architecture of the proposed approach.

Thus, the Discriminant model is able to capture the spatial and temporal dependencies of joint positions, regardless of the class that the sequence of joints describes, meaning that the model can successfully assign hyper-labels to new unseen activities that were not part of the training data, even when their real label is completely different from the original ones. The overall architecture of this approach is provided in Figure 3.

In order to render the recognition more robust, a preprocessing step was performed as in [16]. The action sequences were aligned using a view-invariant transformation. This transformation projected the body keypoints from the original coordinate system to a view-invariant coordinate system.

**Figure 4:**
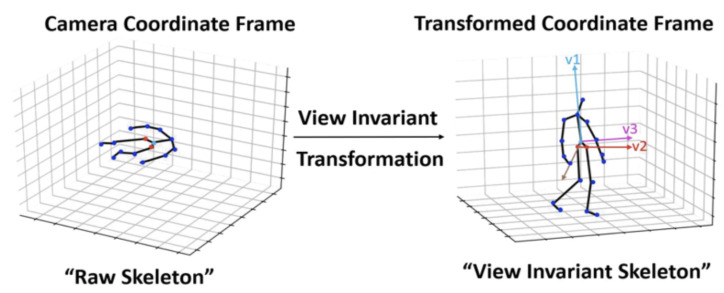
Preprocessing of body keypoints sequences according to view-invariant transformation [16].

Transformation visualization is described in Figure 4, where v1 is the vector perpendicular to the ground, v2 is the difference vector between left and right hips joints in the initial frame of each sequence, and v3 describes the projection into the new space.

The new coordinates of skeleton joints is described by Equation (1):

(1) $$x_{t}^{j}=R^{-1}\left(x_{t}^{j}-d_{r}\right)\forall x,t$$

where $x_{t}^{j}inR^{3x1}$ are the coordinates of the *j*th joint of the *t*th frame, $d_{r}$ is the coordinate of the root joint in the initial frame, and R is the rotation matrix.

The Discriminant model should be able to assign labels in an unsupervised manner; therefore, two clustering algorithms were proposed: K-Means [22] and Gaussian Mixture Models (GMM) [23].

K-Means is an iterative expectation-maximization algorithm used for clustering multidimensional data by using a distance measure function that corresponds to the similarity between two given data points. For the proposed approach, the Euclidean distance was used to compute the similarity of two embeddings in the latent space. The K-Means algorithm begins with the initialization of the k clusters’ centroids in the search space, where k is equal to the number of fixed clusters. This initialization is performed randomly. The expectation step assigns each data to its nearest centroid. Then, the maximization step computes the mean of all the data assigned to each cluster and sets the new centroid. Then, these two steps are repeated until convergence is obtained.

A characteristic of K-means is that it is a hard clustering method, which means that it will associate each point to one and only one cluster. Soft clustering, the GMM [23] approach, unlike K-Means does not use a distance metric to describe the similarity between data points. It is a distribution-based model, which starts from the assumption that the data are distributed in the search space as parts of some Gaussian Distributions; this means that each Gaussian Distribution defines a corresponding cluster of the data. A Gaussian Distribution is described by its mean and its covariance. If we assume that the data are distributed over k clusters, then the GMM should be able to model k Gaussians over the search space such that each data point becomes assigned to one of these Gaussians. This can be written mathematically as a probability of a data point to be generated by a Gaussian.

A GMM involves the mixture of multiple Gaussian Distributions. The probability density function is given by Equation (2):

(2) $$p\left(x\right)=\sum_{k=1}^{K}P\left(k\right)N\left(x|\mu_{k},\sum_{k}\right)$$

where

- P(k) is the mixture proportion of the *k*th distribution. This factor weighs the contribution of the *k*th distribution into the mixture, such that $\sum_{k=1}^{K}P\left(k\right)=1$;
- $N(x|\mu_{k},\sum_{k})$ denotes the conditional probability of the instance x for the *k*th Gaussian distribution. $\mu_{k}$ and $\sum_{k}$ are the mean and covariance of that Gaussian distribution.

Similar to how the K-Means model works, the GMM is also optimized using expectation maximization, which tries to find the Gaussians to maximize the probability of the data points being a part of them.

The advantage of GMM over K-Means is that GMM does not use a distance similarity measure; instead, it is a probabilistic model which attempts to maximize the likelihood of data points to be part of certain Gaussians. Thus, instead of using just the mean of the data, it also uses the variance of it as well.

To summarize, the main contribution of the proposed method consists in a combination of supervised and unsupervised methods. The first part, the Discriminant model, describes an unsupervised manner of grouping up activities based on a similarity measure, using K-Means or GMM over an embedding space of an autoencoder. The second part, the Classifier, utilizes the information obtained by a Discriminant to enhance the results of a baseline supervised classification model (in this case ST-GCN model). Some key improvements will be presented in the following sections.

## 4. Results

The implementation was made using the PyTorch [24] deep learning framework. Both training and evaluation have been executed on a machine equipped with an Nvidia RTX 2080 Ti with 11GB VRAM. The experiments used Adam as the optimization strategy. The loss function used in this work is cross entropy in order to backpropagate gradients. The weight decay is set to 0.0001. All of the training processes ended after 80 epochs.

To train the network, all the skeletal data were preprocessed according to the view-invariant transformation detailed in Section 3 and downsampled to have at most 50 frames. The downsampling mechanism truncates the activity sequence if the number of skeleton frames is greater than 50, and if there are fewer than 50 frames, we padded the sequence with zeros. The truncation is computed using the following equation:

(3) $$frames{}_{downsampling}=frames\left[1+i*diff\right]$$

where the following is the case.

(4) $$diff=\frac{seq_{length}}{target_{frames}}$$

$seq_{length}$ is the total number of frames in each video, $target_{frames}$ is set to 50 throughout the experiment, and *i* = 1 … $target{}_{frames}$

To train the network, all the skeletal data were preprocessed according to the view-invariant transformation and downsampled to have at most 50 frames. The downsampling mechanism truncates the activity sequence if the number of skeleton frames is greater than 50, and if there are fewer than 50 frames, we padded the sequence with zeros.

### 4.1. Datasets

Evaluation of the proposed method was performed on two public datasets: NTU-RGB+D [19] and NTU-RGB+D 120 [25].

NTU-RGB+D [19] is one of the largest multimodality indoor-captured action-recognition dataset. The dataset includes several data modalities, such as RGB videos, IR videos, and skeleton data. It consists of 56.880 videos, which describe 60 different activities. Each video is depicted as a sequence of frames, where each frame contains the 3D world positions for each joint. NTU-RGB+D is a very complex dataset, where the environments in which the scenes are recorded vary in terms of lighting conditions and background variations. The dataset contains RGB videos, depth map sequences, 3D skeleton data, and infrared (IR) samples for each activity sample. The resolution of the videos is 1920 × 1080, while the depth maps and the IR videos are 512 × 424. The skeleton data contains 3D coordinates of 25 joints at each frame.

**Figure 5:**
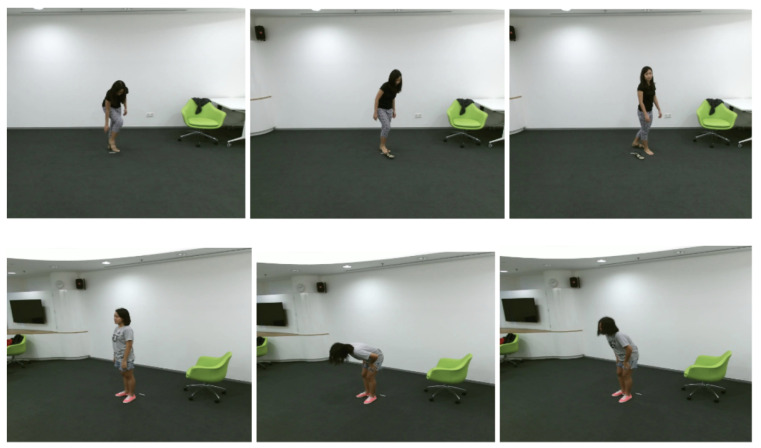
Samples from NTU-RGB+D dataset. Top: A17—take off a shoe; Bottom: A48—nausea/vomiting.

One of the most challenging aspects of this dataset resides in the large amount of classes that it presents. The authors proposed to divide the activities into three major groups. ‘Daily Actions’ represents those types of actions performed in usual scenarios: eat meal, brush teeth, reading, etc. The second group, ‘Medical Conditions’, describes situations strictly related to the unwellness of the human body, and they provide great interest in elderly care: falling down, chest pain, etc. The last group surprises mutual interactions between two people: pushing, giving object, etc. We present some RGB samples from the dataset in Figure 5.

NTU-RGB+D 120 [25] is an extension of the canonical dataset NTU-RGB+D. Over the previous 60 actions dataset, they have added another 60 classes with new subjects recording the videos, with an additional number of 57.600 video clips. This dataset is also divided into three major categories: daily activities and health-related and mutual actions. A few examples from each category are shown in Table 2.

The NTU-RGB+D 120 dataset is, probably, the most descriptive dataset available for the task of HAR. Each video is depicted as a sequence of frames, where each frame contains the 3D world positions for each one of the 25 joints. The subjects in this dataset have various ages ranging from 10 to 57 years. The dataset is governed by realistic variations in terms of the quality of the actions captured. On top of all of them, the environments in which the scenes are recorded vary in terms of lighting conditions and background colors, which results in much larger variations over the entire dataset. The authors, however, provide the full skeleton data from each of the recorded videos. The large number of classes and depicted fine-grained activities renders this dataset appropriate for evaluating the task of Human Activity Recognition.

The cross-subject benchmark splits the 106 actors into two groups, mainly the training group and the testing group. Each group consists of 53 actors.

The cross-view benchmark, on the other hand, splits the dataset based on the setup used to record the video. In this benchmark, the setups identified as even numbers are used for training, while the odd-numbered setups are used for testing.

**Figure 6:**
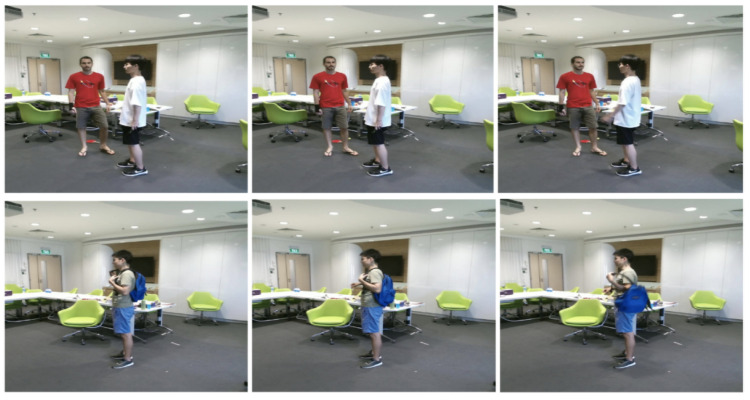
Samples from NTU-RGB+D 120 dataset. Top: A115—take a photo; Bottom: A88—take off bag.

However, most of the state-of-the-art approaches that use this dataset chose another model for splitting the data. The cross-subject evaluation remains the same, but the cross-view evaluation is switched for the cross-view evaluation, where instead of splitting the dataset over the setup identifier, the dataset is split amongst the camera identifiers used for recording. Mainly, the videos recorded by cameras identified as ‘2’ and ‘3’ are used for training, while the remaining ones, videos recorded by camera ‘1’, are used for testing. Figure 6 shows a few samples of this dataset.

### 4.2. Evaluation Results on NTU-RGB-D Dataset

For this dataset, three values were considered for the number of clusters, for both K-Means and GMM methods.

The size of the latent space is equal to 2048, a large value which makes the visualization of the data distribution impossible. We need a method to visualize the data in a space that has fewer dimensions. To solve this issue, we have used a machine learning algorithm called ‘t-distributed stochastic neighbor embedding’ [26] (t-SNE). The t-SNE algorithm begins by computing a probability distribution that resembles the similarity of points. Specifically, the similarity between two points $x_{i}$ and $x_{j}$ is equal to the probability P(*i*|*j*), meaning that the point $x_{i}$ would pick $x_{j}$ as its neighbour, if neighbors are picked to fit a Gaussian centered at $x_{i}$. t-SNE will compute these probabilities in the high dimensional space, the original one, and then it will also compute the same probabilities of the projected points in the 2D space where we want to visualize the data. Then, it will try to minimize the divergence between the two distributions for each point in the data.

The result of t-SNE is a mapping from the high dimensional space into the 2D space of each of the points, meaning that we can clearly visualize the distribution of the points. Indeed, the results are not perfect, but it can provide a method of visualizing the data distribution in a two-dimensional space.

**Figure 7:**
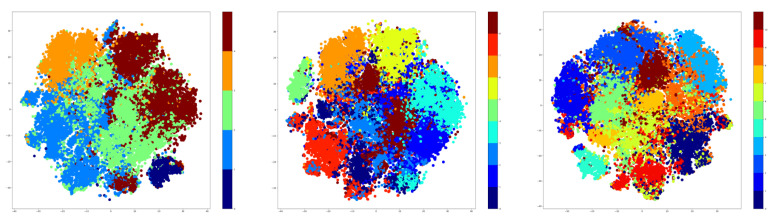
t-SNE plotting for K-Means applied on NTU-RGB+D dataset using 5, 9, and 15 clusters.

**Figure 8:**
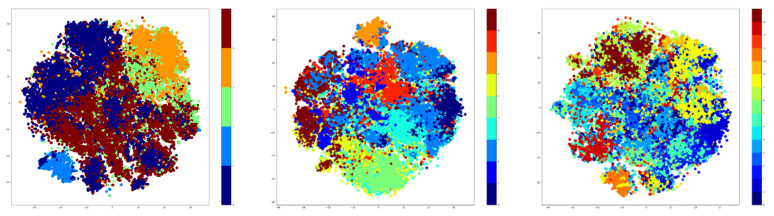
t-SNE plotting for GMM for NTU-RGB+D dataset using 5, 9, and 15 cluster.

Therefore, t-SNE was used to create plots using the embeddings of the training data. For testing, the number of clusters was given three values that were used for both K-Means and GMM methods. These values are as follows: 5, 9, and 15. The t-SNE plots obtained after extracting the embeddings from the training data are shown in Figure 7 for the K-Means algorithm, while the corresponding t-SNE plots are shown in Figure 8 for the GMM algorithm.

The visualization presented previously denotes that the number of clusters is highly dependent on the number of the original classes. Specifically, the seven-cluster K-Means and GMM provide an assignment of hyper-labels that describe a high overlap between the data points, which is natural because there are only 10 original labels. The five-clusters approach provides better results than the seven-clusters, but the most representative result comes from the GMM algorithm using three clusters.

The next step is to train the main architecture from one end to the other. The best configuration of the training parameters is described by the Adam Optimizer with an initial learning rate of 0.001, $\beta_{1}$ = 0.9, and $\beta_{1}$ = 0.999. The batch size used is equal to 512, and the configuration of the ST-GCN is the original one proposed in the original paper, with subsequent ST-GCN layers that increase in dimensions, in order to obtain a higher granularity of the features. The temporal kernel size of the ST-GCN is equal to nine, and the partitioning strategy used for the graph convolution is the spatial one, which groups up joints based on their centripetal and centrifugal properties.

Table 3 shows the results for both the K-Means approach and the GMM approach. The results with the GMM clustering are better, which is expected given the fact that the visualization of the GMM embeddings projects the data in a manner that clearly separates the data in different clusters, unlike the K-Means approach.

The accuracy decreases when the number of clusters increases to 15 based on the probabilistic nature of the clustering models and the latent space size of the autoencoder. The embeddings do not vary that much over individual components, and these variations are incorrectly captured when using a larger number of clusters.

### 4.3. Architecture Improvement

One disadvantage of the proposed method consists in using multiple classifiers. Since the encoder and the clustering algorithm outputs hyper-labels for each activity, it was proposed to use a different approach than using multiple classifiers and activating each of them based on the hyper-label. Thus, the hyper-label becomes an additional feature that is used during the prediction of a single ST-GCN. The idea is to concatenate the output of the ST-GCN before the fully connected layer with the hyper-label assigned by the clustering algorithm. The training mechanism for the 1-Classifier approach is depicted in Algorithm 2.
**Algorithm 2** Algorithm for training of the improved architecture.**N** = number of clusters**E** = encoder model**K-means** = K-means modelSTGCN = one ST-GCN model**for**$X_{seq}$, $Y_{label}$ in dataset **do**    $embeddings\leftarrow E(X_{seq})$    $Y_{pseodo}\leftarrow K-means.predict\left(embeddings\right)$    $X_{seq}\leftarrow concat\left(X_{seq},Y_{pseudo}\right)$    $Y_{pred}=STGCN\left(X_{seq}\right)$    $Error\leftarrow CrossEntropy(Y_{pred},Y_{label})$    BackPropagate Error through STGCN**end for**

The advantage of this method consists in using a single classifier instead of multiple ST-GCN models. The data are extended before the classification part with the ‘hyper-label’ such that activities that are assigned to the same ‘hyper-label’ are weighted together during error propagation through the ST-GCN.

#### 4.3.1. Evaluation of the Improved Model on the NTU-RGB+D Dataset

**Figure 9:**
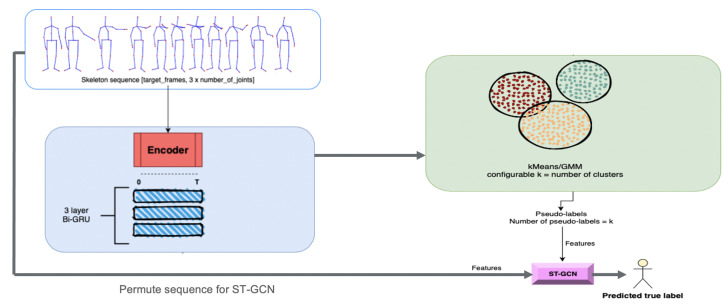
Architecture for 1-Classifier Approach.

The results of the 1-Classifier approach are presented in Table 4, and these results are the best ones from our experiments. The overall architecture is presented in Figure 9.

An analysis of misclassifications produced by the architecture on the NTU-RGB+D dataset was performed. We noticed that the hyper-labels produced by the clustering algorithm are distributed unevenly in the pseudo categories when compared to the ground-truth labels. For instance, the activities from Labels 15 and 23, in the case of a GMM clustering using nine clusters, were distributed as displayed in Table 5. This shows that, for Label 15, 3.52% of the samples would have to be learned by the third classifier, and 7.07% of the samples would have to be learned by the fifth classifier, while the rest of 89.41% of the data would have to be learned by the fourth classifier. The same scenario happens for all the activities, but we picked Labels 15 and Labels 23 as examples, because they are the most representative. The uneven distribution of data hurts the training regime of the architecture as the classifiers cannot generalize well on small distributions due to the lack of information.

In order to fix this problem, a majority mechanism was implemented such that samples belonging to the same class pass through a single classifier. For this part, we have used the ground-truth label to gather all samples belonging to each class. Then, we picked the hyper-label for each sample as the absolute majority of hyper-labels assigned by the clustering algorithm per class. Moreover, this forces the classifiers to only learn a subset of the activities, without learning a small number of examples from other activities which just happened to be clustered by the clustering algorithm in a different cluster.

This technique allowed the architecture to better capture the intrinsic representation of the skeleton sequence, increasing the Top-1 accuracy by more than 5%, as shown in Table 6.

#### 4.3.2. Evaluation of the Improved Architecture on NTU-RGB+D 120 Dataset

The second set of experiments involved the NTU-RGB+D 120 dataset. The extension added 60 more classes, bringing a total of 120 classes. All the samples are performed by 106 subjects in scenes with various illumination settings and different backgrounds. In total, NTU-RGB+D 120 contains 114.480 samples.

The purpose of this experiment is to verify if the proposed architecture is able to generalize on a new set of data. Even though the challenges of the NTU-RGB+D dataset remain to be overcome in this experiment, the extension brings another set of variations of the data that further increase the challenging nature of the task.

The first issue that was encountered was the variations of subjects that are undergoing the activities. The second part of the dataset increased the number of subjects from 40 to 106, resulting in other variations in the body movements due to the individual characteristics of each person. Table 7 describes the results of the extended dataset on our approach.

**Figure 10:**
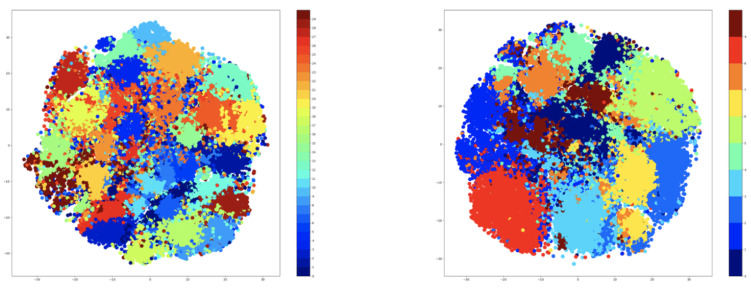
Plotting the t-SNE of encoded features with GMM; 30 clusters (**left**); 10 clusters (**right**).

Based on the results that are shown in Table 7, the retraining of the ‘clustering’ step hurts the performance of the approach on the NTU-RGB+D 120 dataset. The problem comes from the specific encoding of the activities, and it can be observed by plotting the t-SNE features of the hidden space of the encoder. The visualization is available in Figure 10. Unlike the original encoding of the NTU-RGB+D dataset, the new encoding shows undefined clusters, with plenty of overlaps between the samples. In this case, not even the ‘majority’ mechanism can alleviate the problem, because this breaks the distribution of classes across the clusters. Some of the clusters will have fewer classes than the others, resulting in the degradation of the training process.

The next evaluation started from the fact that one of the main issues in supervised methods consists in how the approach can generalize on new data. Moreover, if the new data contains new labels, classification models must be retrained from scratch so as to consider the new labels.

In unsupervised methods, however, the architecture should be able to correctly represent unseen data, making it invariant to additional samples. Keeping this in mind, the extended NTU-RGB+D 120 dataset was used with the ‘clustering’ step untouched (as it was obtained for NTU-RGB+D dataset), without retraining it. The obtained results are given in Table 8.

The analysis undergone for NTU-RGB+D and NTU-RGB+D 120 shows that the 1-Classifier approach is not able to capture the similarities in the embeddings space when combined with the features from the ST-GCN model. The main issue here is described by the nature of the features themselves, as the embeddings are computed in a manner that allows for regeneration of the input sequence in the encoder-decoder model based solely on the recurrent aspect of joint positions in time, while the ST-GCN features are modeled as the main characteristics of the joint positions via the intra-dependency and the interdependency between them.

#### 4.3.3. Data Augmentations

Data augmentations provide a great benefit to neural networks in terms of performance, as various transformations applied on the input data results in a more robust model. Keeping in mind that the input is truncated to a subset of 50 samples, a relatively small number when compared to the initial sequence, the augmentations are meant to improve the quality of the training dataset. However, using too many augmentations might be detrimental to the training process, resulting in the incapability of the network to learn the main characteristics. In this section, we will briefly describe the transformations that have been applied to the skeleton input and the results that we have obtained, along with a short discussion about different augmentation strategies, all while keeping in mind the trade-off between the number of augmentations and the accuracy increase.

Usually, augmentations are used in recurrent based methods, as this trick is crucial when it comes to training accurate models. Papers [27,28,29] proposed a series of transformations to compensate for the lack of training data. Moreover, augmentations are also implemented during the training phase of convolutional neural networks with the objective of simulating various perturbations of the environment (lighting, noise, crops, and occlusion). We propose following a similar direction and implementing different augmentation techniques in our architecture.

During the training phase, we define the transformations as a list of consecutive operations applied on the input data. This list is defined as transf = [$t_{1}$, $t_{2}$, …], where $t_{i}$ is the *i*th augmentation applied on the input sample. Throughout our experiments, each individual transformation is randomly utilized for each sample with a probability equal to 50%.

The first augmentation implemented is shear. Shear’s role is to simulate different perception angles. Thus, each joint’s position is projected in a predefined direction, making the entire skeleton sequence displaced under a certain viewpoint. This augmentation helps the network to capture the small variations of the skeleton movement with respect to the *y* axis.

Flip is the operation that is performed along the temporal dimension, where the skeleton sequence is fed backwards into the neural network, as the model should be capable of understanding the natural series of joints even when they are reversed. Moreover, reversing the input sequence allows the network to better discern between activities that are symmetrical to each other with respect to the temporal axis. We have also applied joint masking where we mask a random number of joints from a random number of frames by replacing them with zeros. This should encourage the model to be more robust to occlusion and able to correctly recognize activities that involve more than one person. We implemented this augmentation in a heuristic fashion where we go through each timestamp in a sequence, we randomly pick a joint (80% chance to not pick any joint), and we randomly set one of the three coordinates to zero (50% chance to not set any coordinate to zero).

The last augmentation technique is represented by Gaussian Noise. Skeleton data are often imprecise and inaccurate, and most of the time the joints’ positions are approximated. However, the model should not be affected by these perturbations; thus, Gaussian noise N(0, 0.05) was added over joints’ position.

**Figure 11:**
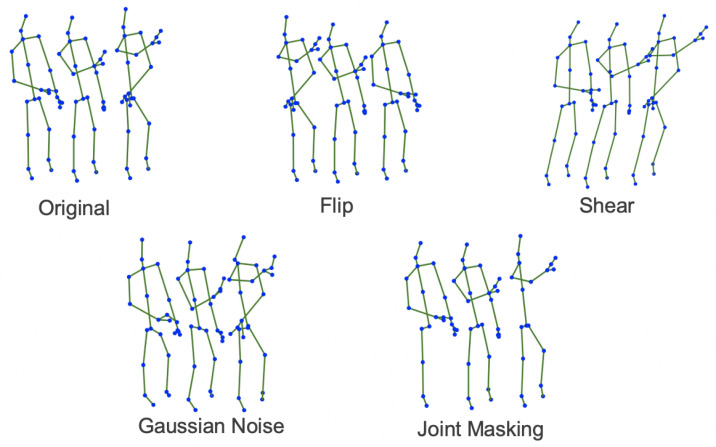
Examples of data augmentation applied on skeleton.

Figure 11 shows a set of data augmentations applied on the human skeleton.

Different experiments were performed with various lengths for the list of augmentations on the NTU-RGB+D and NTU-RGB+D 120 datasets, and the results are given in Table 9.

Different experiments were made using several combinations of data augmentation. First, only one type of data augmentation is applied: flip and shear. Next, experiments were performed by combining flip and shear. Last tests combined flip and shear, and a large set of augmentations were performed, including Gaussian Noise and Joint Masking.

All these experiments were performed on both NTU-RGB+D and NTU-RGB+D 120 datasets. In both cases, the best performances were obtained using flip and shear for data augmentation (an increase in approximately 5% was obtained in both cases of NTU-RGB+D and NTU-RGB+D 120 datasets). In this case, the model can better generalize the activities that were similar to each other in terms of movements of the body, with respect to the vertical axis, while also capturing the difference between reversed sequences of mirrored activities in time. In the case of including Gaussian Noise and the Joint Masking, both performances were reduced since the overall noise induced by all the augmentations hurt the skeleton structure, resulting in ineffective training.

**Figure 12:**
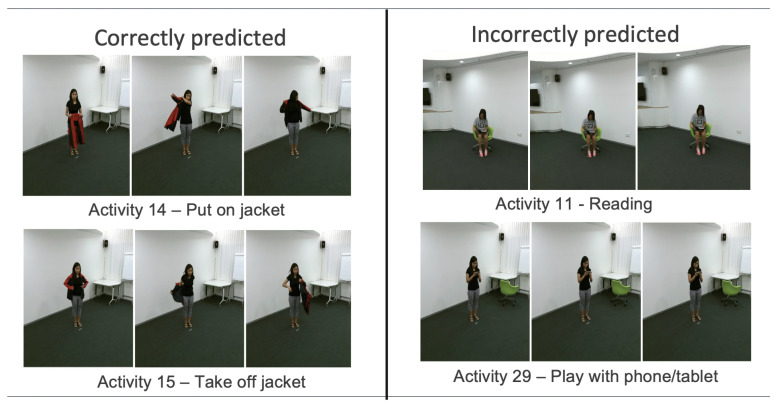
Correctly predicted (**left**) and incorrectly predicted (**right**) examples recognised by the proposed network.

Some examples of good and bad activities (from the point of view of the proposed method) are given in Figure 12.

### 4.4. Performance Analysis of the Improved Model

Relevant information about the performance of the proposed architecture is presented. First, the inference time was analysed. Inference time is the metric that is important in real-life scenarios, as we expect that an application that leverages deep learning techniques should work in real time.

Experiments were performed on a machine equipped with an Nvidia RTX 2080 Ti, having 11 GB of VRAM. We are interested in the execution time between inputting the data into the model and obtaining the output. We report our findings in Table 10.

Table 10 shows that the performance of the clustering method, when compared to the ST-GCN baseline, is almost the same, with indistinguishable increase. The testing of the inference time was performed by using a batch size of 128, both for the ‘clustering’ step and the ‘classification’ step. The duration of the inference over 100 steps was averaged. The addition of the clustering step increased the inference time by more than 150%, but it was expected since the ‘clustering’ step requires passing through the encoder and a prediction using the clustering algorithm, whether it is K-Means or GMM. However, the total time of the inference is less than 5 ms, making the method easily implementable in real life scenarios.

In terms of computational efficiency, the additional computations added by the clustering step were analysed. Even though the number of parameters has increased with a factor of four over the standalone ST-GCN model, the performance is hurt only during the initial training step. The efficiency comes in two large benefits. Firstly, the embedding space of the autoencoder allows new categories of activities to be assigned to existing clusters without the need of retraining the autoencoder. Secondly, the inference time still favors real-time applications, as the clustering requires only a prediction.

Along the reported inference time, some of the misclassified activities were analyzed in order to understand why the architecture is not able to predict the correct label. For example, Table 11 reported our Top-1 accuracy for GMM 9 clusters with Majority for the NTU-RGB+D dataset, where activities labeled 11 (headache) and 37 (wipe face) have an accuracy of 44.44%, respectively 55.70%. In this case, these activities describe very fine movements of the skeleton, with very small variations of the joints’ positions. Due to the large distance between the camera and the subject and because the skeleton is estimated, these small movements act more similar to a jitter, and the network considers them as noise, resulting in misclassification.

On the other hand, the activities ‘put on jacket’ and ‘take off jacket’ are correctly classified most of the time because the movements of the body show clear displacements over time, canceling the possible errors carried by the joints’ information.

### 4.5. Comparison with State of the Art Methods

Table 12 and Table 13 presents the best results of the proposed architectures on the NTU-RGB+D and NTU-RGB+D 120 datasets and also the accuracies of some of the state of the art methods. It can be observed that the proposed method surpasses the performance of the unsupervised approach, fulfilling our objective of proposing a combination of unsupervised and supervised techniques. With respect to Shift-GCN and AngNet [30], the proposed method is not able to reach their performance, but this is caused by the exploitation of the structural data that both these methods imply.

For example, the Shift-GCN model introduces the Shift Graph Spatio Temporal Convolution, specializing the Graph Convolution operation such that it extends the receptive field on both the temporal dimensions and the spatial ones. On the spatial domain, all the nodes share information, unlike the proposed approach where the information is shared between neighboring nodes. Moreover, the temporal domain shows a similar strategy by introducing a learnable parameter related to the quantity of information flow across consecutive frames. These improve the accuracy of the model considerably, and they seem to be a natural extension of our approach, as they are also based on the ST-GCN architecture.

The results obtained for the NTU-RGB+D dataset are similar to the ones obtained by the state-of-the-art methods (as given in Table 12), but for the NTU-RGB+D 120 dataset a decrease in performance is obtained (as given in Table 13), and we alleviate this decrease through the introduction of data augmentations. Moreover, the initial results of the unsupervised method presented in Predict and Cluster were improved, showing that the combination of unsupervised and supervised methods increases the performance when compared to individual implementations.

For the NTU-RGB+D 120 dataset, our results were not optimized entirely, and a better technique would be to use the SGD optimizer instead of Adam, with a corresponding learning rate schedule. Moreover, our approach is not able to correctly capture the large variations between the subjects’ postures and movements as the encoding of the activities is based on the initial set of subjects and their characteristics.

## 5. Conclusions and Future Work

This paper presents a method for human activity recognition by using skeleton data that combines supervised and unsupervised techniques. Several state-of-the-art methods were analysed showing that similar activities are often misclassified due to their similar body movements and postures which could also trick the human eye. The proposed method consists of a two-stage architecture. The first stage groups up activities by using a clustering method over a hidden representation, which better resembles our samples. The second stage classifies activities from each cluster with high granularity of the Ground Truth labels. For the first stage, an encoder-decoder architecture was used to extract the hidden representation of the inputted data. The hidden latent space of the encoder proved to be a suitable candidate for defining a space in which activities are situated at different volumes, with similar activities belonging to the same volume. The distances between activities that are different from each other are much larger than the distances between similar activities, allowing us to use a clustering algorithm to group up similar activities. In the second stage, a classifier was used for each cluster that was created in the first stage. Thus, a classifier does not need to learn the entire dataset but only a subset of the data, meaning the activities that were assigned to its corresponding cluster. On the other hand, adding new activities does not imply a retraining of the entire architecture. The first stage does not require retraining, as the hidden representation will assign the activity to one pre-existing cluster, and in the second stage, the only retraining that must be conducted is the retraining of the corresponding classifier, heavily increasing the retraining time based on the number of activities assigned to that cluster.

The effectiveness of the final architecture was tested on two public datasets: NTU RGB+D and NTU-RGB+D 120. Several experiments with various clustering methods, augmentation techniques, and hyperparameter settings were conducted. The proposed model achieved 90.2% Top-1 Accuracy on NTU-RGB+D dataset and 74.06% Top-1 Accuracy on NTU-RGB+D 120 dataset. These results proved to be more efficient than unsupervised techniques and even improved the baseline model (ST-GCN).

As future work, the baseline ST-GCN that takes the role of the classifier from the second stage can be replaced with stronger architecture such as the Shift-GCN, which better captures the temporal and spatial correlation of the joint positions, all while keeping the number of parameters in the same magnitude order. On the other hand, since the number of classes of a dataset increases, an exploration through a larger latent space might yield improved results as more dimensions in the latent space might result in greater disentanglement of the intrinsic feature representations.

## Figures and Tables

**Table 1:** Results of the state of the art methods on NTU-RGB+D.

Methods	Top-1 Accuracy	
	C-View	C-Subject
ST-GCN [15]	88.3%	81.55%
Predict and Cluster [16]	74.82%	50.21%

**Table 2:** Examples of activities from NTU-RGB+D 120 dataset.

Category	Classes
Daily Actions	Open bottle, fold paper, and make victory sign
Health-related	Blow nose, stretch oneself, and yawn
Mutual Actions	Exchange things, high-five, and rock-paper-scissors

**Table 3:** Results of the Discriminative Approach.

Clustering Algorithm	Number of Clusters	Initial Learning Rate (Adam Optimizer)	Batch Size	Accuracy (%)	Training Time (min)
K-Means	5	0.005	128	74.05	230
K-Means	9	0.005	128	76.03	235
K-Means	15	0.005	128	74.72	239
GMM	5	0.005	128	78.33	247
GMM	9	0.005	128	79.56	250
GMM	15	0.005	128	76.39	260

**Table 4:** Results of the 1-Classifier Approach.

Clustering Algorithm	Number of Clusters	Initial Learning Rate (Adam Optimizer)	Batch Size	Accuracy (%)	Training Time (min)
K-Means	9	0.005	128	85.72	181
GMM	9	0.005	128	87.26	193

**Table 5:** Uneven distribution of hyper-labels.

	Cluster 1	Cluster 2	Cluster 3	Cluster 4	Cluster 5	Cluster 6	Cluster 7	Cluster 8	Cluster 9
Label 15	0%	0%	3.52%	89.41%	7.07%	0%	0%	0%	0%
Label 23	0%	0%	0%	0%	0%	98.21%	0%	1.79%	0%

**Table 6:** Results of the majority technique.

Clustering Algorithm	Number of Clusters	Initial Learning Rate (Adam Optimizer)	Batch Size	Accuracy (%)	Training Time (min)
GMM (with Majority)	9	0.005	128	85.24	254

**Table 7:** Results of the NTU-RGB+D 120 dataset.

Clustering Algorithm	Number of Clusters	Initial Learning Rate (Adam Optimizer)	Batch Size	Accuracy (%)	Training Time (min)
GMM (with Majority)	10	0.005	128	41.33	515
K-Means (with Majority)	10	0.005	128	40.33	509
GMM (with Majority)	30	0.005	128	39.41	561
K-Means (with Majority)	30	0.005	128	36.97	547
GMM with 1-Classifier	10	0.005	128	23.15	413

**Table 8:** NTU-RGB+D 120 results with 60 classes encoding.

NTU-RGB+D 120 Results with 60 Classes Encoding	Number of Clusters	Initial Learning Rate (Adam Optimizer)	Batch Size	Accuracy (%)	Training Time (min)
GMM (with Majority)	9	0.005	128	67.87	450
K-Means (with Majority)	9	0.005	128	59.29	441
GMM (with Majority)	15	0.005	128	63.21	498
K-Means (with Majority)	15	0.005	128	51.07	449
GMM with 1-Classifier	10	0.005	128	23.15	413

**Table 9:** Top-1 accuracy with different augmentations.

Methods	Augmentations	Initial l.r. (Adam)	Batch Size	Accuracy (%)	Dataset
GMM—9 clusters	No augmentations	0.005	128	67.87	NTU-RGB+D 120
GMM—9 clusters	Flip	0.005	128	72.31	NTU-RGB+D 120
GMM—9 clusters	Shear	0.005	128	72.78	NTU-RGB+D 120
GMM—9 clusters	Flip + Shear	0.005	128	74.06	NTU-RGB+D 120
GMM—9 clusters	Flip + Shear + Gaussian Noise + Joint Masking	0.005	128	56.03	NTU-RGB+D 120
GMM—9 clusters	No augmentations	0.005	128	85.24	NTU-RGB+D
GMM—9 clusters	Flip	0.005	128	86.40	NTU-RGB+D
GMM—9 clusters	Shear	0.005	128	88.04	NTU-RGB+D
GMM—9 clusters	Flip + Shear	0.005	128	90.22	NTU-RGB+D
GMM—9 clusters	Flip + Shear + Gaussian Noise + Joint Masking	0.005	128	71.58	NTU-RGB+D

**Table 10:** Performance results of the proposed approach.

Approach	Dataset	Clustering Step (ms)	Classification Step (ms)	Total (ms)	Number of Parameters
ST-GCN—baseline	NTU-RGB+D	N/A	0.71	0.71	4.54 × $10^{5}$
Clustering (K-Means 9) (Ours)	NTU-RGB+D	1.90	0.70	2.60	1.7 × $10^{6}$
Clustering (GMM 9) (Ours)	NTU-RGB+D	2.11	0.71	2.82	1.7 × $10^{6}$
Clustering (GMM 9) Majority (Ours)	NTU-RGB+D	2.03	0.72	2.75	1.7 × $10^{6}$
Clustering (GMM 10) Majority (Ours)	NTU-RGB+D 120	2.18	0.71	2.89	1.7 × $10^{6}$
Clustering (GMM 9) Enc-60 (Ours)	NTU-RGB+D 120	2.13	0.71	2.84	1.7 × $10^{6}$

**Table 11:** Top-1 Accuracy GMM 9 clusters with majority for few examples from NTU-RGB+D dataset.

Activity	Top-1 Accuracy (%)
Label 3—brush teeth	93.04
Label 5—drop	90.19
Label 7—throw	96.20
Label 8—sit down	96.19
Label 9—stand up	99.05
Label 14—put on jacket	99.37
Label 15—take off jacket	98.73
Label 2—eat meal	62.66
Label 11—reading	44.44
Label 37—wipe face	55.70
Label 29—play with phone/tablet	24.37

**Table 12:** Comparison of the proposed approach with state of the art methods (NTU-RGB+D dataset).

Approach	Dataset (X-Sub)	Top-1 Accuracy (%)
ST-GCN—baseline	NTU-RGB+D	81.5
Clustering (K-Means 9) (Ours)	NTU-RGB+D	76.0
Clustering (GMM 9) (Ours)	NTU-RGB+D	79.5
Clustering (GMM 9) Majority (Ours)	NTU-RGB+D	85.2
Clustering (GMM 9) Majority with augmentations (Ours)	NTU-RGB+D	90.2
Shift-GCN [31]	NTU-RGB+D	90.7
Predict and Cluster [16]	NTU-RGB+D	50.7
AS-GCN [15]	NTU-RGB+D	86.8
AngNet + BA + JBA + VJBA [30]	NTU-RGB+D	91.7
VPN++ [32]	NTU-RGB+D	96.7

**Table 13:** Comparison of the proposed approach with state of the art methods (NTU-RGB+D 120 dataset).

Approach	Dataset (X-Sub)	Top-1 Accuracy (%)
Clustering (GMM 10) Majority (Ours)	NTU-RGB+D 120	41.3
Clustering (GMM 9) Enc-60 (Ours)	NTU-RGB+D 120	67.8
Clustering (GMM 9) enc-60 with augmentations (Ours)	NTU-RGB+D 120	74.0
Shift-GCN [31]	NTU-RGB+D 120	87.6
AngNet + BA + JBA + VJBA [30]	NTU-RGB+D 120	88.2
VPN++ [32]	NTU-RGB+D 120	90.7

## Data Availability

Not applicable.
